# Predictive Performance and Optimal Cut-Off Points of Blood Pressure for Identifying Arteriosclerosis among Adults in Eastern China

**DOI:** 10.3390/ijerph18178927

**Published:** 2021-08-25

**Authors:** Lei Yu, Jiaxiang Yan, Chen Yang, Yanan Gao, Aiwen Wang, Huiming Huang

**Affiliations:** 1 Faculty of Sport Science, Research Academy of Grand Health, Ningbo University, Ningbo 315211, China; llisport@163.com (L.Y.); jiaxiang0016@163.com (J.Y.); gaoyanan19971225@163.com (Y.G.); 2 Department of Kinesiology and Physical Education, McGill University, Montreal, QC H2W 1S4, Canada; chen.yang4@mail.mcgill.ca

**Keywords:** blood pressure, brachial-ankle pulse wave velocity, optimal cut-off point, arteriosclerosis

## Abstract

This study aimed to assess the predictive performance and establish optimal cut-off points of blood pressure for identifying arteriosclerosis in eastern Chinese adults. Brachial–ankle pulse wave velocity (baPWV) was utilized to evaluate arteriosclerosis. The predictive performance of blood pressure for arteriosclerosis was determined by the area under the curve (AUC) of receiver operating characteristics; the optimal blood pressure cut-off points were determined by Youden’s index. A logistic regression model was used to acquire the odds ratio (OR) of blood pressure for arteriosclerosis. The AUCs of blood pressure for identifying arteriosclerosis were 0.868 (95%CI: 0.860–0.875) for systolic blood pressure (SBP) and 0.835 (95%CI: 0.827–0.843) for diastolic blood pressure (DBP), both *p* < 0.01. The AUCs of women were higher than that of men (0.903 vs. 0.819 for SBP; 0.847 vs. 0.806 for DBP; Z test *p* < 0.05). The AUCs in the 18–39.9-years group were higher than that in the 40–59.9-years and 60–84-years groups (0.894 vs. 0.842 and 0.818 for SBP; 0.889 vs. 0.818 and 0.759 for DBP; Z test *p <* 0.05). The total optimal cut-off points of blood pressure for predicting arteriosclerosis were 123.5/73.5 mmHg (SBP/DBP) overall; 123.5/73.5 and 126.5/79.5 mmHg for women and men, respectively; and 120.5/73.5, 123.5/76.5, and 126.5/75.5 mmHg for 18–39.9-years, 40–59.9-years, and 60–84-years groups, respectively. Blood pressure indexes had a high predictive performance for identifying arteriosclerosis with the optimal cut-off point of 123.5/73.5 mmHg (SBP/DBP) in eastern Chinese adults. Women or the younger population have a higher predictive performance and lower cut-off points to identify arteriosclerosis.

## 1. Introduction

Cardiovascular diseases (CVD) are the main cause of death across the world. Arteriosclerosis and atherosclerosis are the causes of most cardiovascular diseases, which are both indicators of future cardiovascular events and intervention targets [1,2]. Arteriosclerosis reflects arterial stiffness and function; furthermore, atherosclerosis indicates pathological changes in the arterial structure, which is often associated with cardiovascular events such as arterial rupture and blockage. According to the report “China cardiovascular diseases report 2018”, cardiovascular diseases occupied the leading cause of death, accounting for 44% of all deaths in China [3]. This leads to a heavy burden on medical care resources and the economy in China.

At present, pulse wave velocity is a classic index for the noninvasive detection of arteriosclerosis [4,5]. Brachial–ankle pulse wave velocity (baPWV) is the conduction velocity of the pulse wave at a certain distance from the arterial segment (i.e., pulse wave velocity), which represents the elasticity and compliance of arterial blood vessels [6,7,8,9]. BaPWV has increasingly been used as an epidemiological investigation method for diagnosing arteriosclerosis. Earlier screening and intervention for arteriosclerosis are vital for reducing disability and cardiovascular mortality [10]. Relatively speaking, the measurement of baPWV is complex and expensive. In contrast, blood pressure is easy to measure, and it has consistently been demonstrated that blood pressure is closely associated with arteriosclerosis [11,12,13]. However, what threshold value of blood pressure should be controlled to prevent arteriosclerosis is still unclear.

The predictive performance and accurate warning cut-off point of blood pressure can help patients to take earlier actions to prevent arteriosclerosis. Although we know that there is a close correlation between blood pressure and arteriosclerosis, the predictive performance and appropriate sex- and age-specific cut-off points of blood pressure for identifying arteriosclerosis have not been reported in Chinese adults. Therefore, this study aimed to assess the predictive performance and to develop sex- and age-specific blood pressure cut-off values for identifying arteriosclerosis.

## 2. Materials and Methods

### 2.1. Study Population

This study was designed as a cross-sectional study. We collected 9830 medical records from five cities in eastern China (Nanjing, Ningbo, Suzhou, Changzhou, and Taizhou) from 2015 to 2019. Height, weight, age, systolic blood pressure (SBP), diastolic blood pressure (DBP), heart rate, smoking and exercise status, and baPWV were the variables of interest. In order to minimize the confounding effects from antihypertensive drugs, we excluded 215 subjects with a hypertension history who currently were taking antihypertensive drugs and with blood pressure controlled to a normal range (SBP < 140 mmHg and DBP < 90 mmHg). Finally, we recruited 9615 qualified subjects aged 18 to 84 years, with 4766 women and 4849 men. This study was conducted in accordance with the Declaration of Helsinki. All the participants in this study signed an informed consent form. This study followed the principles outlined in the Declaration of Helsinki and was approved by the Institutional Review Board of the Faculty of Sports Science, Ningbo University.

### 2.2. Measurements

Basic information about participants was collected by professionals with questionnaires. The same measurement tools and protocol were adopted in each of the five cities.

Blood pressure measurement: After a 5-min rest, the seated brachial blood pressure of each participant was measured using a mercury sphygmomanometer with standard calibrations. BaPWV measurement: The baPWV was measured using an oscillometer-based device (BP-203RPE; Omron-Colin, Japan) by a trained technician. The subjects rested for at least 5 min in the supine position, with four cuffs wrapped around the upper arm and ankle, and then the arteries of the upper and lower extremity were measured simultaneously via a noninvasive shock pressure wave graph.

### 2.3. Definitions

Arteriosclerosis: Blood vessel elastic function was assessed by baPWV. Arteriosclerosis was defined by a baPWV ≥ 1400 cm/s [14,15,16]; hypertension: participants taking antihypertensive drugs or SBP ≥ 140 mmHg or DBP ≥ 90 mmHg; blood pressure grade: normal blood pressure, with SBP < 120 mmHg and DBP < 80 mmHg; high–normal blood pressure: SBP in the range of 120–139 mmHg and/or DBP in the range of 80–90 mmHg; grade 1 hypertension: SBP in the range of 140–159 mmHg and/or DBP in the range of 90–99 mmHg; grade 2 hypertension: SBP in the range of 160–179 mmHg and/or DBP in the range of 100–109 mmHg; grade 3 hypertension: SBP ≥ 180 mmHg and/or DBP ≥ 90 mmHg [17].

Smoking status: smokers were defined as those who smoked at least one cigarette per day continuously or cumulatively for 6 months [18]; exercise status: exercisers were defined as those who exercised through at least 30 min of moderate-intensity physical activity three times per week (60% to 90% of maximum heart rate reserve or 50% to 85% of maximum oxygen intake) [19,20]; Body Mass Index (BMI) = weight(kg)/height (m)^2^ [21].

### 2.4. Statistical Analysis

Statistical analysis was performed with SPSS software (version 25.0). Continuous variables (height, weight, BMI, resting heart rate, SBP, DBP, and baPWV) were presented as the mean ± standard deviation (SD); comparisons among groups were conducted by a one-way ANOVA. Comparisons of rates (arteriosclerosis%, hypertension%, smoker%, and exerciser%) were performed by a chi-square test. The predictive performance of blood pressure was evaluated by the area under the curve (AUC) of receiver operating characteristics (ROC) for identifying arteriosclerosis. Comparisons of AUCs between the two groups were conducted by a Z-test. The optimal cut-off points on the ROC curve were determined by the maximal Youden’s index (sensitivity + specificity − 1). Crude odds ratios (ORs), as well as adjusted ORs, were calculated by a multiple logistic regression model to analyze the association between blood pressure and arteriosclerosis. Adjusted factors included age, sex, resting heart rate, BMI, smoker, and exerciser, among which sex, smoking, and exercise status were treated as categorical variables. The percent attributable risk of the population (PARP) was calculated by the exposure percentage and ORs, based on the optimal cut-off points of blood pressure for identifying arteriosclerosis. The formula is given as follows: PARP = 100 × *p* (OR − 1)/[*p* (OR − 1) + 1]%.

## 3. Results

### 3.1. Baseline Characteristics of Subjects

A total of 9615 subjects were included in this study. Height, weight, BMI, heart rate, SBP, DBP, baPWV, and smoking and exercise statuses were collected. Of the included participants, 4849 were men, and 4766 were women; their ages ranged from 18 to 84 years. A small portion of the records on smoking and exercise were not collected in the questionnaire, and missing subjects therein were ignored. The Kolmogorov–Smirnov test showed that all the continuous variables (height, weight, BMI, resting heart rate, SBP, DBP, and baPWV) had a normal distribution, all of which were *p* > 0.05. There were differences in baseline measurements in sex and age groups, *p* < 0.01 (a one-way ANOVA test for continuous variables; a chi-square test was used for categorical variables). The characteristics of the subjects are shown in Table 1.

### 3.2. Prevalence of Arteriosclerosis in Different Blood Pressure Grades

Among the 9615 subjects, 312 subjects with normal blood pressure had arteriosclerosis, with a prevalence of 6.9%. All of the 66 subjects with grade 3 hypertension had arteriosclerosis, with a prevalence of 100%. The prevalence of arteriosclerosis was raised with the increase in the blood pressure grade (*p* < 0.01). The relationships between blood pressure grades and the prevalence of arteriosclerosis are shown in Table 2.

### 3.3. Predictive Performance and the Optimal Blood Pressure Cut-Off Points

**Figure 1:**
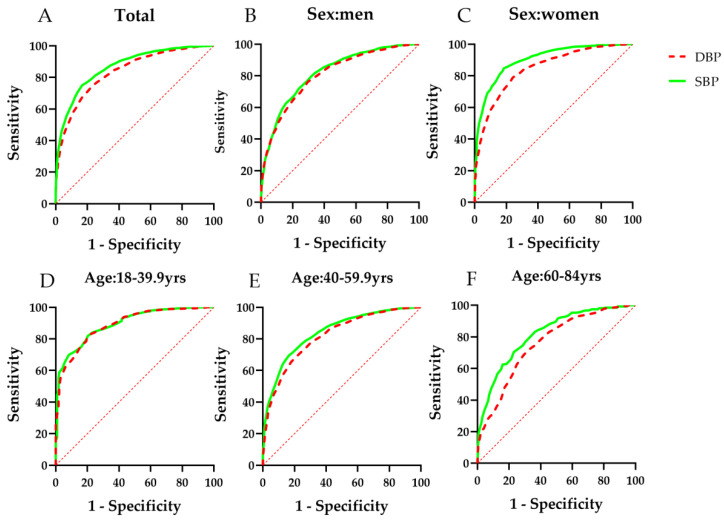
ROC curves of blood pressure for identifying arteriosclerosis in Chinese adults. (**A**) total subjects; (**B**) subjects in men; (**C**) subjects in women; (**D**) subjects aged 18 to 39.9 years; (**E**) subjects aged 40 to 59.9 years; (**F**) subjects aged 60 to 84 years; ROC, receiver operating characteristic; SBP, systolic blood pressure; DBP, diastolic blood pressure; yrs, years.

The AUC values of sex- and age-specific blood pressure for identifying arteriosclerosis were 0.868 for SBP and 0.835 for DBP, both of which had effective predictive performance (*p* < 0.01). The AUC values of both SBP and DBP for women for identifying arteriosclerosis were higher than those for men (Z test, *p* < 0.01). As age increased, the predictive performance of blood pressure for identifying arteriosclerosis decreased (Z test, *p* < 0.01). The details are shown in Figure 1 and Table 3.

The optimal cut-off points of sex- and age-specific blood pressure for identifying arteriosclerosis were 123.5/75.5 mmHg (SBP/DBP), and the corresponding sensitivity/specificity reached 0.832/0.756 for SBP and 0.768/0.747 for DBP. According to their Youden’s index values, women and young groups had a higher predictive performance. The optimal cut-off points of SBP and DBP for men were higher than those for women; meanwhile, the cut-off points of the older group were slightly higher than those of the younger group (Table 4).

### 3.4. ORs in the Multiple Logistic Regression Model and PARP on Arteriosclerosis

The AUC values of sex- and age-specific blood pressure for identifying arteriosclerosis were 0.868 for SBP and 0.835 for DBP, both with effective predictive performance (*p* < 0.01). The multivariate logistic regression model on arteriosclerosis was adjusted by age, sex, resting heart rate, BMI, smoking status, and exercise status. Compared with the subjects below the optimal blood pressure cut-off points (shown in Table 4), the crude OR value of those who had a higher blood pressure was 15.887; meanwhile, the adjusted OR value decreased significantly to 8.845. With regard to the features of sex or age, the relative OR values of women were larger than those of men, while the OR values decreased gradually with an increase in age. According to the adjusted OR and exposure percentage, the total PARP of blood pressure was 70.6% (sex- and age-specific PARP reached 70.1% and 70.0% in women and men, respectively, and 50.2%, 75.8%, and 84.9% in 18–39.9-years, 40–59.9-years, and 60–80-years groups, respectively). The details are listed in Table 5.

## 4. Discussion

Arteriosclerosis measured by baPWV was a strong predictor of cardiovascular mortality [13,22,23]. Thus, the early detection of arteriosclerosis or arterial stiffness could help to assess the risk stratification and reduce the prevalence of cardiovascular events. In the past decade, studies have investigated the relationships between blood pressure and arteriosclerosis or high arterial stiffness, measured by pulse wave velocity in different populations worldwide [24,25]. Studies in Japan found that SBP, mean arterial pressure (MAP), and arterial stiffness are closely related [26,27]; Wang et al. found that increased blood pressure was the main factor affecting arteriosclerosis in Chinese adults [28]. Arteriosclerosis has multiple risk factors, such as age, sex, blood pressure, blood glucose, lifestyle, etc. [26,28]. Due to the convenience of measuring blood pressure, assessing the predictive performance of blood pressure for arteriosclerosis could facilitate screening arteriosclerosis earlier. Therefore, our aims were to assess predictive performance and to establish the optimal cut-off points for blood pressure in identifying arteriosclerosis in the Chinese population. The prevalence of hypertension changes significantly with age and sex. Accordingly, we stratified the data by age and sex and developed the sex- and age-specific predictive performance of blood pressure. We retained samples from all ages so that we could analyze the characteristics of age.

The prevalence of arteriosclerosis was high in our study because it is more sensitive to diagnosis under the same vessel condition. As blood pressure levels increased, the prevalence of arteriosclerosis also gradually increased. Our result, that there was a positive correlation between blood pressure and arteriosclerosis, is consistent with previous studies [26,27,28]. It is widely known that many factors, such as age, gender, family history of coronary artery disease, hyperlipidemia, smoking, eating habits, etc., can affect both hypertension and arteriosclerosis. Therefore, these factors could be considered potential confounders when studying the association between blood pressure and arteriosclerosis. For example, the percentage of smokers was very different among different sex and age groups in the baseline of this study, which may have led to potential confounding bias in the sex- and age-specific associations between blood pressure and arteriosclerosis. In general, a stratified analysis can be used to deal with confounding bias. When multiple potential confounding factors exist, a multiple logistic regression model is a better statistical method, due to the advantage of controlling the mixed relationship between multiple factors. Therefore, we used a multiple logistic regression model to control the confounding factors of sex, age, BMI, smoking and exercise status for analyzing the association between blood pressure and arteriosclerosis. However, in this study, some CVD risk factors, such as hypercholesterolemia and hyperglycemia, were not considered due to the lack of blood biochemical indicators, which may have overestimated the effects of ORs in the multiple logistic regression model and the effects of PARP. Our main results regarding the predictive performance and optimal cut-off points of blood pressure were relatively less affected by these potential confounding factors because the AUCs of the ROC curve and the cut-off points for identifying arteriosclerosis are stable and independent. In addition, we also considered the effects of hypertension drugs. Pharmacological treatment can affect blood pressure and, thus, would be a confounding factor in a blood pressure study. Wilkinson et al. have suggested that studies should start in youth to minimize the initial confounding effects of arteriosclerosis and pharmacological treatment [29]. In order to minimize the confounding effects of antihypertensive drugs, we excluded 215 subjects with a hypertension history who were currently using antihypertensive drugs and whose blood pressure was controlled in the normal range.

Generally, the predictive performance, which ranges from 0.5–1, is determined by the AUC of the ROC curve. An area that exceeds 0.8 indicates good predictive performance. The results of the AUCs indicated that two kinds of blood pressure indexes have a high predictive performance for arteriosclerosis. The AUCs of SBP and DBP to assess arteriosclerosis were 0.868 and 0.835, respectively. Although blood pressure can predict arteriosclerosis well, it may be more accurate to predict arteriosclerosis when combined with other indicators [6]. The optimal cut-off points of SBP/DBP for predicting arteriosclerosis were 123.5/75.5 mmHg, respectively. The clinical diagnostic standard of hypertension is 140/90 mmHg, while the optimal blood pressure cut-off points calculated in our study for predicting arteriosclerosis were lower than the diagnostic standard. If the clinical diagnostic standard of hypertension is applied to predict arteriosclerosis, many subjects with arteriosclerosis will be erroneously diagnosed. Our results of blood pressure cut-off points were close to the diagnostic values for those with high–normal blood pressure (120/80mmHg for SBP/DBP), which indicates that people with high–normal blood pressure have a higher risk of arteriosclerosis. A cohort study considered a blood pressure cut-off threshold of <122.5/76 mmHg as being optimal for discriminating cardiovascular mortality; meanwhile, the cut-off point of <135/83 mmHg for blood pressure has an independent prognostic value of cardiovascular mortality [30]. Another cohort study recommended 130/80 mmHg as the optimal cut-off threshold in self/home blood pressure measurements [31]. Our research indicates that even if blood pressure does not meet the diagnostic standard for hypertension (140/90 mmHg for SBP/DBP), when the SBP or DBP exceeds 123.5 or 75.5 mmHg, respectively, people also are exposed to the risk of encountering arteriosclerosis.

In the current study, the predictive performance of blood pressure for identifying arteriosclerosis in sex and age was different. The AUCs for SBP/DBP (0.902/0.846) were higher in women than in men (0.819/0.806), which indicates that the predictive performance for women would be more accurate and also revealed a closer relationship with arteriosclerosis in women. Further analysis through multiple logistic regression showed that the OR value of women (11.4) was greater than that of men (6.2), which was adjusted by age, resting heart rate, BMI, and smoking and exercising status. Although women, in general, have a lower absolute prevalence of arteriosclerosis, when women with a higher blood pressure are defined by the optimal cut-off threshold, they will have a higher relative risk of arteriosclerosis due to the higher OR value. Therefore, we suggest that women should pay attention to the risk of arteriosclerosis as their blood pressure increases. Pertaining to age characteristics, the younger groups showed a higher predictive performance of blood pressure for identifying arteriosclerosis due to higher AUCs. Moreover, as age increased, the adjusted OR values showed a decreasing trend from 13.8 to 10.4 to 8.5, which indicates that young people with high blood pressure have a higher relative risk of arteriosclerosis. It is well known that hypercholesterolemia is one of the main determinants of arteriosclerosis. Thus, it is notable that the above OR values may be overestimated due to the lack of data on the prevalence of hypercholesterolemia and hypertriglyceridemia in the multiple logistic regression model. Tuscarora et al. showed that advanced age and being male are associated with a higher baPWV [32]. Ai et al. found that age has an influence on baPWV, the effect of which is more obvious in women [33]. Although there is compelling evidence to support the fact that being male or aging are significant risk factors for arteriosclerosis [26,27,28,29], the association between blood pressure and arteriosclerosis was stronger in women or the younger population. As a result, women or the younger population might have a higher predictive performance and lower cut-off points for identifying arteriosclerosis. In addition, our study also implied that there may be interaction on arteriosclerosis between blood pressure, age, and sex-based on the sex- and age-specific OR values calculated by the logistic regression analysis. Further multiplicative or additive interaction statistics might contribute to the analysis of the interaction between multiple factors with arteriosclerosis. The results of this study suggest the importance of the early prevention and treatment of hypertension in reducing the risk of arteriosclerosis. The target population for the prevention of arteriosclerosis is not only limited to patients with hypertension. Those who have high–normal blood pressure should also be concerned with the risk of arteriosclerosis. According to the maximal Youden’s index (sensitivity + specificity − 1) of the ROC curve, we determined the corresponding optimal sex- and age-specific cut-off points of blood pressure for identifying arteriosclerosis among adults in China. Women or the younger population have lower cut-off points for both SBP and DBP than men or the older population. Based on these results, we calculated the PARP according to adjusted ORs and exposure percentages. The epidemiological significance of the PARP analysis showed that if intervention measures are taken to control blood pressure below the optimal cut-off points (123.5 mmHg for SBP and 75.5 mmHg for DBP), 70.6% of arteriosclerosis would be prevented across the whole population. If blood pressure is controlled under 123.5/73.5 mmHg for women, 126.5/79.5 mmHg for men, 120.5/73.5 mmHg for 18–39.9 years, and 126.5/75.5 mmHg for 60–84 years, 50.2–84.9% of arteriosclerosis would be prevented.

At present, instruments to measure baPWV are complex and expensive; therefore, this method is mainly applied in large hospitals in China. However, blood pressure can easily be measured in the ordinary population in any primary hospital or community in China. Thus, the application of the predictive performance of blood pressure to identifying arteriosclerosis could help doctors make better decisions and suggestions. Overall, our study showed that blood pressure has a high predictive performance for arteriosclerosis.

## 5. Conclusions

Blood pressure indexes had a high predictive performance for identifying arteriosclerosis with the optimal cut-off point of 123.5/73.5 mmHg (SBP/DBP) in eastern Chinese adults. Women or the younger population have a higher predictive performance and lower cut-off points to identify arteriosclerosis.

### Limitations

There are some limitations to our study. The present study is cross-sectional design, which cannot be used to establish temporal relationship and causality; thus, the validation of optimal cut-off points needs further prospective cohort studies as support. Second, a lack of data on prevalence of hypercholesterolemia and hypertriglyceridemia in the multiple logistic regression model may lead to an overestimation of ORs and PARP. Finally, the subjects were from five cities in eastern China, so the study’s findings cannot necessarily be representative of all Chinese adults.

## Figures and Tables

**Table 1:** Baseline characteristics by sex and age in the subjects.

Variables	Sex			Age (Yrs)	
	Women (*n* = 4766)	Men (*n* = 4849)	18–39.9 (*n* = 2901)	40–59.9 (*n* = 5581)	60–84 (*n* = 1133)
Height, cm	159.2 ± 5.5	170.3 ± 6.1 ^a^	165.5 ± 8.1	165.1 ± 7.9	161.9 ± 8.2 ^a^
Weight, Kg	59.0 ± 8.5	72.6 ± 10.1 ^a^	63.4 ± 12.4	67.3 ± 11.1	65.1 ± 10.7 ^a^
BMI, Kg/m^2^	23.3 ± 3.1	25.0 ± 3.0 ^a^	23.0 ± 3.4	24.6 ± 3.0	24.8 ± 3.1 ^a^
HR, beats/min	72.1 ± 9.8	73.0 ± 11.5 ^a^	72.7 ± 10.4	72.5 ± 10.4	72.6 ± 11.0
SBP, mmHg	117.8 ± 17.1	127.2 ± 15.3 ^a^	114.1 ± 13.4	124.6 ± 16.1	134.2 ± 18.5 ^a^
DBP, mmHg	69.5 ± 10.8	77.3 ± 10.7 ^a^	66.5 ± 9.7	76.2 ± 11.0	77.5 ± 10.1 ^a^
BaPWV, cm/s	1262.1 ± 242.6	1377.8 ± 232.7 ^a^	1163.3 ± 160.8	1343.8 ± 207.6	1608.1 ± 283.9 ^a^
Arteriosclerosis, % (*n*)	22.4 (1067)	38.7 (1876) ^b^	7.9 (228)	33.4 (1862)	75.3 (853) ^b^
Hypertension, % (*n*) Smoker, % (*n*) Exerciser, % (*n*)	10.8 (514) 2.6 (105) 22.8 (910)	19.9 (962) ^b^ 38.3 (1573) ^b^ 18.1 (741) ^b^	4.1 (119) 15.8 (388) 15.9 (387)	17.7 (989) 22.7 (1069) 22.0 (1031)	32.5 (368) ^b^ 23.0 (221) ^b^ 24.5 (233) ^b^

Note: values are the means ± SD or % (*n*); ^a^ one-way ANOVA test, *p* < 0.01; ^b^ chi-square test, *p* < 0.01. Abbreviations: BMI, body mass index; yrs, years; HR, heart rate; SBP, systolic blood pressure; DBP, diastolic blood pressure; baPWV, brachial–ankle pulse wave velocity.

**Table 2:** Prevalence of arteriosclerosis in different blood pressure grades.

Arteriosclerosis	Normal Blood Pressure	High–Normal Blood Pressure	Grade 1 Hypertension	Grade 2 Hypertension	Grade 3 Hypertension
Positive, *n*	312	1441	859	237	64
Negative, *n*	4209	2185	283	25	0
Prevalence, %	6.9%	39.7% ^a^	75.2% ^a^	90.5% ^a^	100% ^a^
*p* value ^a^	-	<0.01	<0.01	<0.01	<0.01

Notes: ^a^ chi-square test; the normal group was used as a reference, *p* < 0.01.

**Table 3:** AUC values of sex- and age-specific blood pressure for identifying arteriosclerosis.

Variables	SBP (95%CI)	DBP (95%CI)
Total	0.868 (0.860–0.875)	0.835 (0.827–0.843)
Sex		
Women Men	0.903 (0.893–0.913) 0.819 (0.807–0.831) ^a^	0.847 (0.834–0.860) 0.806 (0.793–0.818) ^a^
Age(years)		
18–39.9 yrs	0.894 (0.875–0.913)	0.889 (0.869–0.909)
40–59.9 yrs	0.842 (0.832–0.853) ^b^	0.818 (0.807–0.829) ^b^
60–84 yrs	0.818 (0.790–0.846) ^b^	0.759 (0.728–0.790) ^b^

Note: ^a^ compared with women, Z test, *p* < 0.05; ^b^ compared with 18–39.9 years, Z test, *p* < 0.05. Abbreviations: SBP, systolic blood pressure; DBP, diastolic blood pressure; yrs, years; AUC, area under the curve.

**Table 4:** Optimal cut-off points, sensitivity, and specificity for blood pressure to identify arteriosclerosis.

Variables	Optimal Cut-Off Points (mmHg)		Sensitivity		Specificity		Youden’s Index	
	SBP	DBP	SBP	DBP	SBP	DBP	SBP	DBP
Total	123.5	75.5	0.832	0.768	0.756	0.747	0.588	0.515
Sex								
Women	123.5	73.5	0.815	0.0.758	0.848	0.790	0.663	0.548
Men	126.5	79.5	0.761	0.703	0.719	0.763	0.480	0.466
Age(years)								
18–39.9 yrs	120.5	73.5	0.798	0.781	0.818	0.829	0.616	0.610
40–59.9 yrs	123.5	76.5	0.836	0.793	0.694	0.683	0.530	0.476
60–84 yrs	126.5	75.5	0.770	0.678	0.704	0.719	0.474	0.395

Abbreviations: SBP, systolic blood pressure; DBP, diastolic blood pressure; yrs, years.

**Table 5:** ORs (95% CI) in the multiple logistic regression model and PARP of arteriosclerosis based on optimal cut-off points for blood pressure.

Variables	Crude OR (95%CI)	Adjusted OR (95%CI)	PARP
Total ^a^	15.887 (14.036–17.981)	8.845 (7.694–10.168)	70.6%
Sex ^b^			
Women	20.022 (16.541–24.237)	11.429 (9.116–14.330)	70.1%
Men	9.030 (7.837–10.404)	6.243 (5.340–7.300)	70.0%
Age(years) ^c^			
18–39.9 yrs	25.480 (15.152–36.604)	13.759 (8.379–20.042)	50.2%
40–59.9 yrs	12.749 (10.666–15.239)	10.367 (8.618–12.472)	75.8%
60–84 yrs	8.940 (6.449–12.394)	8.512 (6.294–11.510)	84.9%

Notes: ^a^ adjusted OR: adjusted for age, sex, resting heart rate, BMI, smoking status, and exercise status; ^b^ adjusted OR: adjusted for age, resting heart rate, BMI, smoking status, and exercise status; ^c^ adjusted OR: adjusted for sex, resting heart rate, BMI, smoking status, and exercise status. Abbreviations: yrs, years; OR, odds ratio; CI, confidence interval; PARP, population attributable risk proportion.

## Data Availability

The data presented in this study are available on request from the corresponding author.

## Abbreviations

ROC	receiver operating characteristic
AUC	area under the curve
OR	odds ratio
PARP	percent attributable risk of population
SBP	systolic blood pressure
DBP	diastolic blood pressure
baPWV	brachial–ankle pulse wave velocity
yrs	years
HR	heart rate
CI	confidence interval
BMI	body mass index
